# Time-varying spectral power of resting-state fMRI networks reveal cross-frequency dependence in dynamic connectivity

**DOI:** 10.1371/journal.pone.0171647

**Published:** 2017-02-13

**Authors:** Maziar Yaesoubi, Robyn L. Miller, Vince D. Calhoun

**Affiliations:** 1 The Mind Research Network, 1101 Yale Blvd NE, Albuquerque, New Mexico, United States of America; 2 Deptartment of ECE, MSC01 1100, 1 University of New Mexico, Albuquerque, New Mexico, United States of America; University of Texas at Austin, UNITED STATES

## Abstract

Brain oscillations and synchronicity among brain regions (brain connectivity) have been studied in resting-state (RS) and task-induced settings. RS-connectivity which captures brain functional integration during an unconstrained state is shown to vary with the frequency of oscillations. Indeed, high temporal resolution modalities have demonstrated both between and cross-frequency connectivity spanning across frequency bands such as theta and gamma. Despite high spatial resolution, functional magnetic resonance imaging (fMRI) suffers from low temporal resolution due to modulation with slow-varying hemodynamic response function (HRF) and also relatively low sampling rate. This limits the range of detectable frequency bands in fMRI and consequently there has been no evidence of cross-frequency dependence in fMRI data. In the present work we uncover recurring patterns of spectral power in network timecourses which provides new insight on the actual nature of frequency variation in fMRI network activations. Moreover, we introduce a new measure of dependence between pairs of rs-fMRI networks which reveals significant cross-frequency dependence between functional brain networks specifically default-mode, cerebellar and visual networks. This is the first strong evidence of cross-frequency dependence between functional networks in fMRI and our subject group analysis based on age and gender supports usefulness of this observation for future clinical applications.

## Introduction

Connectivity analysis has proven to be an important vehicle to study functional integration of brain. Such analysis utilizes a measure of dependence within or among the identified spatial regions of the brain which can either be defined based on prior anatomical knowledge, in the form of regions-of-interest (ROI), or using data-driven approaches such as independent component analysis (ICA).

Based on the observation that brain oscillations, captured with high temporal resolution modalities such as magnetoencephalography (MEG) and electroencephalography (EEG), contain spectral power over a wide range of frequencies including theta (4-7Hz), alpha (7-14Hz), beta (14-25Hz) and gamma (low: 30-60Hz, high: 60-100Hz), as well as evidence of interactions between different frequency bands being related to higher level functions of the brain such as memory management and cognition [1–3], frequency variation of connectivity has been explored in the form of cross and in-between frequency dependence along with temporal dependence such as Pearson’s correlation coefficient as a commonly used domain to measure dependence.

Frequency domain measures of dependence include but are not limited to coherence [4–6]and cross-frequency dependence (or, alternatively, cross-frequency coupling (CFC)). Variations of CFC have been investigated in the form of phase-amplitude coupling, referred as cross-frequency modulation (cfM) [7], phase-phase coupling, referred as phase synchronization [8], and amplitude-amplitude coupling [9]. Particularly, phase-synchronization demonstrated better identification of the underlying cortical connectivity for visual working memory in a combined EEG/MEG study [10]. Further non-linear dependence methods and their applications to EEG/MEG analysis were reviewed in [11] and [12].

Compared to EEG/MEG, the range of detectable frequencies in typical blood oxygenation level dependent [13] fMRI is fairly limited. Recent studies found evidence of frequency variation of BOLD signal interactions [14, 15]. However these intersections have only been observed within specific frequency bands and there is still no evidence of cross-frequency interactions, though we might suspect the existence of such interactions since the BOLD signal is a correlate of actual brain oscillations as well as underlying brain function.

In this work, we observe that: first, the frequency content of rs-fMRI networks activity is dynamic in time, and second, that this variation occurs with respect to multiple patterns of spectral powers rather than being specialized to specific sub-bands. These observations lead us to the design of a novel metric for measuring CFC in rs-fMRI data.

Brain networks, along with their associated time-courses, are captured by independent component analysis (ICA) of fMRI voxel time-series. Estimation of the instantaneous power spectra of network time-courses is achieved by a time-frequency decomposition. The collection of instantaneous network timecourse spectra are then summarized into a small set of recurring spectral states by applying k-means clustering to time-varying network spectra from all subjects. The cluster centroids define canonical patterns of spectral distributions, which we call “Frequency modes”. Without making any prior assumption on the properties of these modes, we observe different spectral density shapes emerging naturally from the data while simple band-pass filtering is unable to capture such information.

Taking a step further, we analyze the occurrence rate of each mode in the original network time-courses, as well as the co-occurrence of pairs of modes, which we use to define a new measure of cross-frequency dependence. Significant gender and age effects are observed with both the occurrence and co-occurrence measures for some specific networks and network pairs, respectively.

We conclude this study by pointing out that although fMRI data suffers from low sampling rate and also being a hemodynamically mediated signal with only a narrow range of detectable frequencies relative to other modalities, there are detectable dynamics and cross-frequency dependencies in this limited range of detectable frequencies which have not been explored before. Significant age and gender associations illustrate the richness of this kind of information.

## Material and methods

We used resting-state scans of 405 all healthy subjects with average age of 21 and ranging from 12–35 years. 200 of the subjects were females. Scans had a minimum duration of 5 min and 4 seconds which with repetition time (TR) of 2 s results in 152 volumes. Excess volumes of subjects with a longer duration were discarded. Also the first 4 volumes were discarded to avoid T1 equilibration effect. Subjects were given written informed consent following institutional guidelines at the University of New Mexico and it was approved by Institutional Review Board of the University of New Mexico. Initial number of subjects was 603 from which subjects maximum translation greater than 1.5mm or subjects with spatial correlation to EPI template less than 0.93 were discarded. All subjects were instructed to keep their eyes open.

### fMRI acquisition and pre-processing

Same scanning device, a 3-T Siemens Trio scanner with and 12-channel radio frequency coil, was used for all the subjects. Gradient-echo EPI sequence with echo time (TE) of 29 ms and repetition time (TR) of 2 s was used. Other scanning parameters were flip angle = 75° with slice thickness of 3.5 mm^3^ and slice gap of 1.05 mm. The field of view was 240 mm and voxel size was 3.75x3.75x4.55 mm^3^.

Standard pre-processing pipeline of SPM 5 (http://www.fil.ion.ucl.ac.uk/spm/software/spm5) was used for pre-processing of the functional images. It includes realignment, slice-timing correction, spatial normalization to Montreal Neurological Institute space and reslicing, and finally a Gaussian kernel was used for spatial smoothing (*σ* = 2.123*mm*).

### Group ICA and post-processing

Group spatial ICA was used to decompose the voxel-level time series into functional brain networks and corresponding time-courses. GIFT toolbox (http://mialab.mrn.org/software/gift) was used for ICA decomposition. Initially, the data was decomposed into 100 networks (ICA components) which include both cortical and subcortical regions. For this, first PCA was used for the initial reduction of subject-level data which retained 120 components for each subject. Then these reduced data were concatenated for all subjects followed by a second PCA which retained 100 group components. Last, Infomax ICA [16] ran on this reduced data with 10 times repetition to find 100 maximally-independent components. GICA1 backreconstruction algorithm was used for the estimation of both subject-specific components and time-courses[17].

Not all these components represent intrinsic functional networks (ICNs) and instead could be linked to motion, physiological and imaging artifacts. Post-processing steps were taken to identify such components including identifying components not having peak activation in grey matter or components with time-courses which did not have considerable low-frequency power. After these steps we are left with 50 ICNs which are visually represented in S1 File.

Finally, time-courses were de-trended(linear, quadratic and cubic) followed by outlier removal as proposed in [18] and also motion parameters were regressed out of time-courses. More details on these post-processing steps can be find in [19] and [20].

### Estimation of instantaneous power spectra of network time-courses

To capture recurring frequency modes we used time-frequency decomposition of network time-courses which enables us to capture frequency content as a function of time. Wavelet analysis is one of the most commonly used and well-established method to achieve such decomposition. Mathematically speaking wavelet kernel is defined as:

$$\psi_{s,\tau }(t)=\frac{1}{\sqrt{s}}\Psi (\frac{t-\tau }{s})$$ (1)

where *s* is scale of the wavelet representing its target frequency and *τ* is the amount of translation of the mother wavelet Ψ from the time origin. Please refer to [21] for more comprehensive tutorial on wavelet decomposition.

In this study we want the explicit notion of frequency so we chose complex Morlet kernel defined as 12πσe2πifcte−t22σ2 which has a Normal distribution in frequency domain. *f*_*c*_ is the center of its frequency distribution and *σ* is variance of the distribution (is set here to 0.01 Hz) and *t* represents time-point. The center frequencies are chosen as uniformly spaced points in the interval of [0–0.25 Hz] which is the detectable range of frequency with sampling rate of TR = 2 seconds.

Since we want to compare wavelet decomposition of the network-time courses between different network and subjects and also we want to make our estimation less susceptible to random variation of signal due to noise artefacts, in the actual implementation, we used z-score estimation of the wavelet decomposition of a given network time-course of each subject. The null distribution of such decomposition comes from a time-series with zero frequencies meaning having no periodic behavior. Here, such null-distribution is derived from bootstrap resampling of the same time-course when its time-points are randomly shuffled to remove any periodic behavior of the signal. This is repeated for 200 times per each network time-course of each subject and wavelet decomposition of these subject-specific shuffled time-courses is estimated. Wavelet estimations of each frequency and over all time-points constitute null-distribution corresponding to that frequency for the given subject.

We now use these z-scored time-frequency decompositions of all network time-courses of all subjects to find the dynamic and recurring frequency modes. To better understand this process, note that each network time-course of each subject has now a 2-d representation. In one dimension there is frequency information and the other is the time dimension. If for a given subject, we concatenate these 2-d representations along the time dimension and for all networks we will have a matrix which one dimension represents the frequency information and the other one has the time information for all networks (*time* x *network*). We further concatenate these subject-specific matrices for all subjects along the time dimension which again forms a 2-d matrix whose one dimension is frequency power spectral and the other dimension is *time* x *network* x *subjects*.

**Fig 1:**
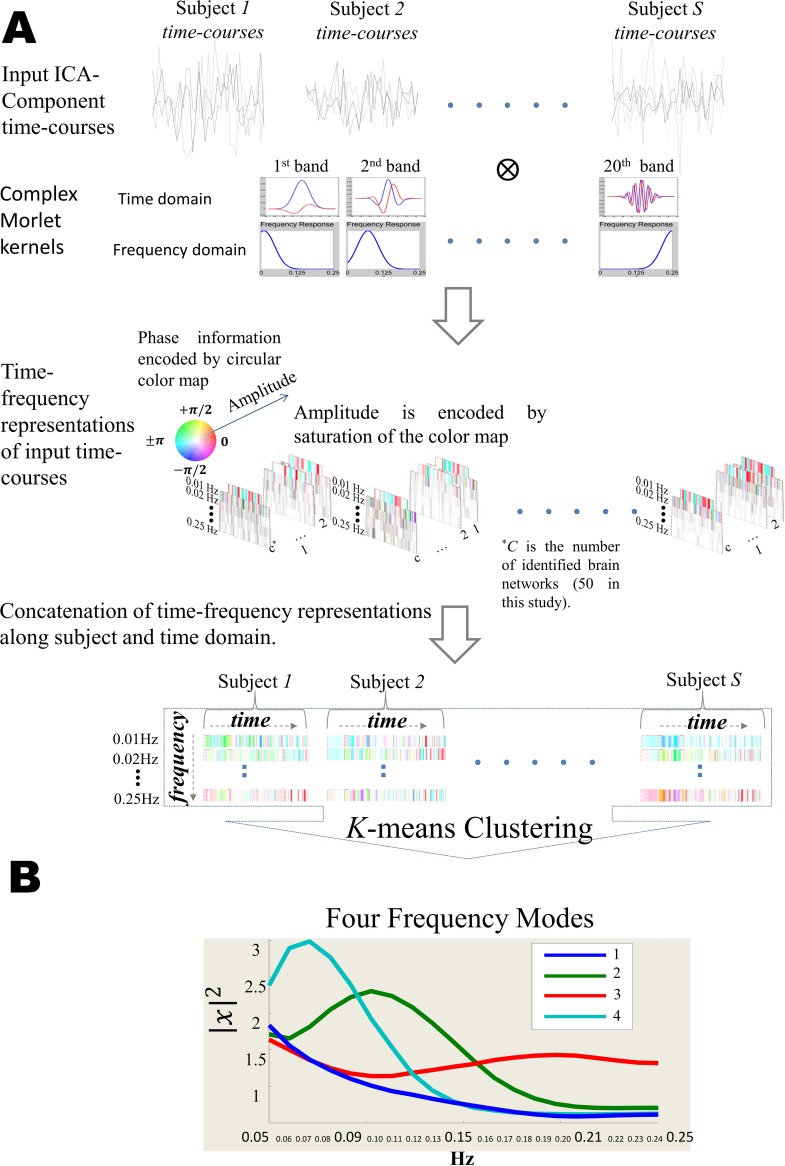
Outline of our framework for capturing instantaneous spectra of ICA time-courses and its variation in time in the form of “frequency modes”. (A) First, fMRI time-series is pre-processed and feed into the ICA to be decomposed into 50 ICA networks and the associated time-courses (detail of these ICA networks is provided in supplementary material of (Allen, 2014 #510)). Complex morlet wavelet is used to map the time-courses to the time-frequency domain. Finally, canonical patterns of power spectra are estimated by k-means clustering which we refer to as “frequency modes”. (B) "Frequency modes" as the representatives of the variation in spectral powers of networks time-courses, Each mode is formed by similar instantaneous frequency content of time-courses which have been clustered together.

Clustering of this matrix along the time dimension (for all networks and subjects) would capture dynamic and recurring frequency modes. In this work we used k-means clustering with k = 4. Fig 1 summarizes all these steps along with the captured frequency modes as the result of the proposed method. Number of clusters (‘k’) is chosen based on the elbow criteria by measuring the ratio of variance in each cluster to the variance between clusters. The elbow curve is shown in S2 File.

## Results

Frequency modes are, in fact, centroids of clusters that are formed by instantaneous spectral powers of all ICA time-courses. The modes represent short-lived and periodic activities of time-courses. The occurrence rate of these activities forms the corresponding spectral densities as is presented in Fig 1B.

Frequency modes, by design, are shared among subjects and networks and F-ratio analysis suggests that 4 of these shared modes explain most of the variations of spectral powers. However, there are different aspects of the modes which may vary between subjects and networks. First, frequency modes vary with respect to the range of frequencies they span. For example, while mode 4 spans a relatively narrow range of frequencies comparing to other modes and its spectral density has positive skewness (left-modal), mode 2, on the other hand, spans middle-range of frequencies with the peek around 0.1 Hz and mode 3 has more uniform spectral density along all range of frequencies and with a slight negative skewness (right-modal) and a peek at 0.20Hz.

**Fig 2:**
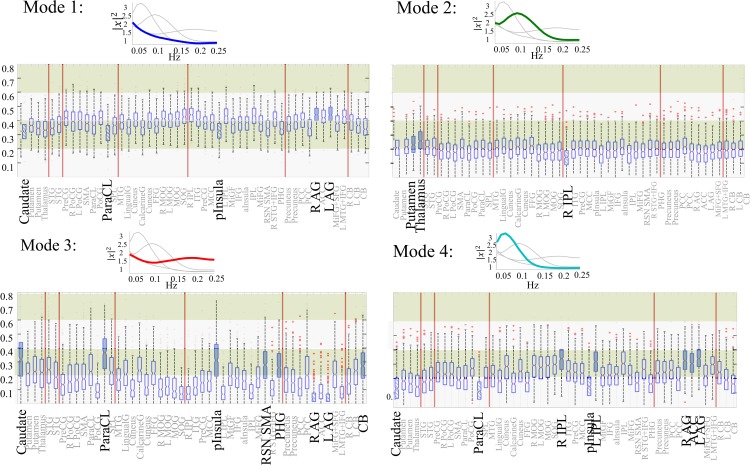
Analysis of occurrence the modes. Boxplots of occurrence rates of each individual frequency mode in ICA networks. Networks with significantly higher (filled boxplots) or lower (dashed boxplots) occurrence of the given mode than majority (85%) of all networks are identified.

Next, we investigate two other aspects of the modes which vary among subjects and networks. The first aspect is the occurrence rate of individual modes in each of the networks which is measured by counting number of time-points of a given network time-course which has been assigned to a certain mode as the result of clustering (Fig 2). Occurrence rate of a mode in a given network, when normalized, represents probability that the given network at any point of time has the frequency profile of the given mode.

For the second aspect of the frequency modes, we investigate dependence between the occurrences of the modes by introducing conditional co-occurrence (***c***co-occurrence) of these modes. As mentioned earlier, since each mode has a unique spectral density, by investigating co-occurrence between each pair of modes, we are in fact exploring a measure of cross-frequency dependence of ICA time courses. Conditional co-occurrence is defined here as a conditional probability of occurrence of a specific mode (assume mode ***i***) in a given network (network ***m***) given that another mode (let it be mode ***j***) has occurred at the same time-point in another network (network ***n***) both belong to the same subject.

Mathematically speaking we measure:

Cco−occurrence(modesi,jinnetworksm,n)=Pr⁡(modeiinnetworkm|modejinnetworkn) (2)

If occurrence of mode ***i*** in network ***m*** is independent of the occurrence of mode ***j*** in network ***n*** then we expect the ***c***co-occurrence as defined above to be equal to the individual occurrence of mode ***i*** in network which means *Cco* – *occurrence*(*modes* ***i*,*j*** *in networks* ***m*,*n***) being equal to Pr(*mode* ***i*** *in network* ***m***).

Consequently any evidence of *Cco* – *occurrence* being unequal to the indivual occurrence of the mode ***i*** provides an evidence of dependence between the two modes. If there is enough evidence that Pr(*mode* ***i*** *in network* ***m*** | *mode* ***j*** *in network* ***n***) > Pr(*mode* ***i*** *in network* ***m***) it means that occurrence of mode ***j*** in network ***n*** has positive effect on the occurrence rate of mode ***i*** in network ***m***. We name such dependence “reinforcement effect”.

Similarly Pr(*mode* ***i*** *in network* ***m*** | *mode* ***j*** *in network* ***n***) < Pr(*mode* ***i*** *in network* ***m***) provides evidence of negative effect of mode ***j*** in network ***n*** on mode ***i*** in network ***m*** which we call it “suppression effect”.

Consequently, our reported estimation of cco-occurrence is *z*-score estimation of above cco-occurrence measure from estimated distribution of Pr(*mode* ***i*** *in network* ***m***) which would serve as our null distribution. As mentioned above, values around zero imply an independence of occurrence of mode ***i*** from mode ***j*** in the given the networks. Values greater than zero implies positive dependence of mode ***i*** on mode ***j*** and negative dependence when the value is less than zero.

**Fig 3:**
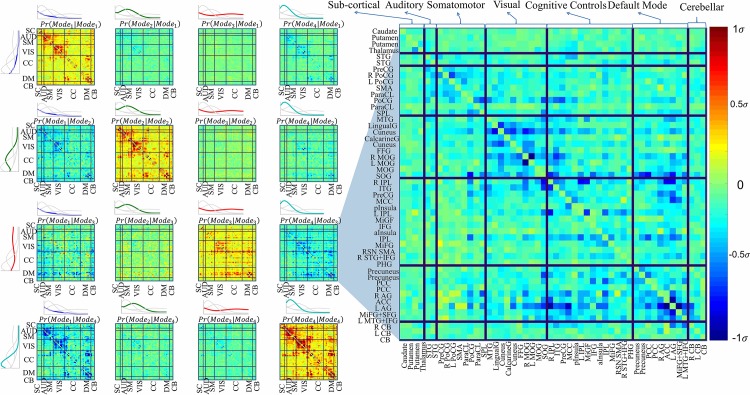
Analysis of co-occurrence rates of the modes. Cco-occurrence maps of frequency mode pairs. An entry (column *m*, row *n*)(1…50, 1…50) of a matrix at column *i* (1…4) and row *j* (1…4) of the figure shows cco-occurrence of frequency mode *i* in network *m*, given that frequency mode *j* is occurred at the same time-point in network *n*. Positive cc-occurrence (color coded as red) corresponds to *reinforcement effect* and negative cc-occurrence (color coded as blue) is corresponding to *suppression effect*.

Fig 3 represent cco-occurrence of each pair of modes for all networks as a *C* x *C* matrix where *C* is number of ICA-networks (50 in this study). We call these matrices “cco-occurrence maps”.

In the last part of the results, we focus on studying variation of individual occurrence of modes as well as cco-occurrences of pair of modes between networks due to the age and gender of subjects.

Variation in occurrence rate of modes between networks can be investigated by boxplot representation of the rates from all subjects for each network. For each mode we identify networks which have significantly higher or lower average occurrence rates than majority of all networks. For this analysis, first, we performed pairwise 2-sample t-test between all network pairs. The estimated p-values were adjusted to account for the multiple comparisons using the false discovery rate (FDR) approach. Second, we identified networks which had significantly (FDR adjusted p-value < 0.01) higher (solid filled box-plot in Fig 2) or lower (dashed box-plot in the same figure) average occupancy rates than 80% of all networks. S2 File provides more detailed descriptions of identified networks.

We also study the effect of age and gender of subjects on the estimated occurrence rate of modes through a multivariate analysis of covariance by setting the occurrence rate of each mode in all the networks (a 50-d vector for each mode) as the response variable. The design matrix includes age, gender and two motion parameters (average translation and rotation of each subject) as well as pair-wise interactions of these predictors. This followed by a backward selection of the subset of significant predictors. Backward selection implementation of MANCOVAN toolbox (http://www.mathworks.com/matlabcentral/fileexchange/27014-mancovan) was used to perform this procedure. For more details on the multivariate analysis please refer to [19].

**Fig 4:**
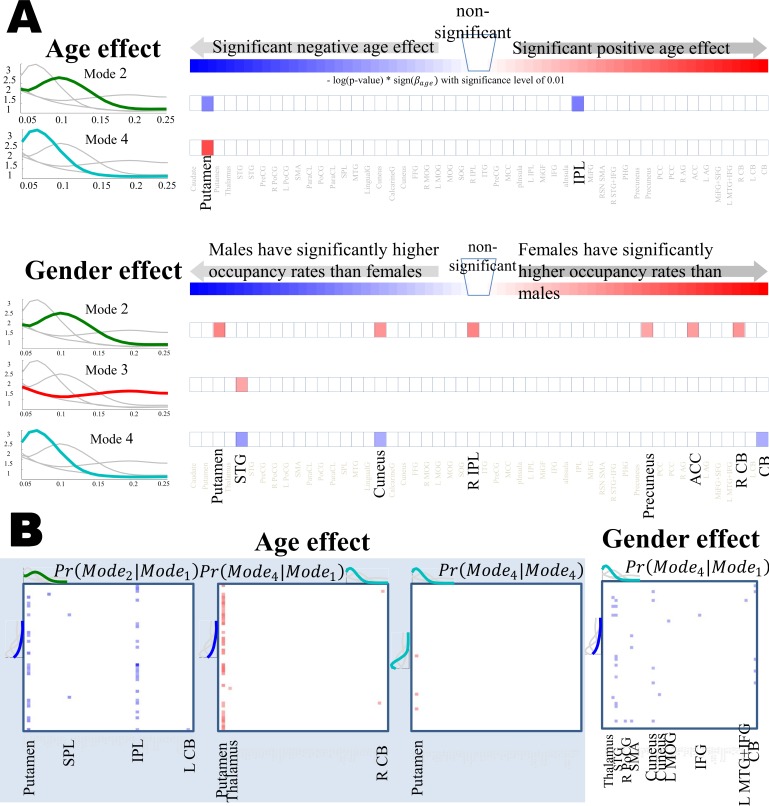
Analysis of age and gender effect on. (A) occurrence rate of individual frequency modes and (B) cco-occurrence rate of pair of modes. In (A) specific networks and in (B) pairs of networks are highlighted [13] in which occurrence rate of given mode and cco-occurrence of pair of modes are significantly effected by age or gender

The multivariate analysis determines the subset of predictors which significantly affect occurrence rate of each mode over all networks. If the reduced model included either age or gender as significant predictor we can identify individual networks in which given mode’s occurrence rate is significantly affected by either of those predictors. Univariate analysis for each individual network is used for this part. First, predictor of interest is picked (which is either age or gender if included in the reduced model). Then the effect of other predictors in the model is regressed out from occurrence rate of the given mode in individual networks through a linear regression. Finally, a linear regression is applied between the selected predictor and the residual of the previous regression as the response variable. After running this for all networks, the estimated p-values are FDR adjusted for the multiple comparisons and the ones which survived the significance level of 0.01 are reported. Fig 4A shows −log(FDR-Adj *p-value*) × *sign*(regression coefficient) for networks with significant age and gender effect on mode occurrence rate. The sign of the regression coefficient shows direction of influence. For age effect it shows if the measure is increased or decreased by age and for gender, positive coefficient means the measure of interest is higher in females since female is encoded as ‘1’ and male as ‘0’.

A similar analysis can be used to study variation of cco-occurrence of frequency modes between networks and subjects. Each cco-occurrence map, by itself, shows variation among networks. For example, from the maps along diagonal which represent dependence between same modes, we are observing that although there is relatively higher dependence between all networks comparing to the observed dependence in the other maps, dependence within somatomor and visual networks is stronger.

To study subject-related variation of co-occurrence maps due to age and gender of subjects, we follow similar analysis to the one we used for the occurrence rate of individual modes. The design matrix would stay the same and the response variable would be the cco-occurrence maps of each pair of modes. Same as before, Fig 4B shows −log(FDR-Adj *p-value*) × *sign*(regression coefficient) for pairs of networks in which cco-occurrence of given pairs of modes is significantly affected by age and gender. Interpretation on the directionality of the influence and the color maps for p-values stays the same as in Fig 4A.

## Discussion

This study adds to the growing evidence that, although fMRI data has limited range of detectable frequencies, even within that narrow range of frequencies, connectivity varies both within and across frequencies.

In this work, by employing “frequency modes” that capture characteristic spectral power distributions, frequency variation is observed in individual network time-courses. Conditional co-occurrence of these modes is our measure of cross-frequency dependence between brain networks.

There are interesting observations that can be made based on cco-occurrence maps in Fig 2B. First, positive cco-occurrences observed in diagonal maps which correspond to cco-occurrence of same frequency modes, resembles familiar modularity in resting state functional connectivity including connectivity between Default Mode (DM), Visual (VIS) and Somatomotor (SM) networks. In fact, occurrence of a given mode in each of these networks reinforce occurrence of the same mode in those other networks. We call this effect a “reinforcement effect”. Continuing with this observation, we observe that any reinforcement effect between a pair of components on the diagonal is accompanied by negative (which we call “suppression”) cco-occurrence in off-diagonal maps. Specifically, suppression effect on Mode 1 by other modes observed at column one has a direct interpretation. Mode 1 has lower power at every frequency than any of the other modes, representing very weak signals with spectral power that decays in frequency. Between network-pairs that tend to show high correlation in conventional FNC analyses, the two modes with highest aggregate power (i.e. modes 2 and 4) seem to self-reinforce, while both separately suppress the probability of seeing mode 1 with low-magnitude spectrum with decaying frequency. This leads us to conclude that networks with strong signals selectively reinforce networks with similar frequency regime while also suppressing the odds that such a network presents relatively weak signal.

Other than reinforcement effect among same modes (diagonal maps of Fig 3) and suppression effect on mode 1 by other modes (first column of the same figure), if there did not exist any dependence across frequency modes, we should not have observed any "selective" suppression or reinforcement effect between any pair of modes (please refer to S3 File for simulation results based on conventional BOLD signal generation model). A good example of such independence can be observed on rows 2 and 4 of the figure. In these rows, other than reinforcement effect on the diagonal and suppression effect on mode 1 by either of the modes (2 and 4), there is no significant effect on any other remaining modes. S4 File further confirms that such observed independence is consistent with the cco-occurrence observed under the true independence forced by the conducted simulations as is explained in detail.

However on row three of the figure, we observe a suppression effect by mode 3 on mode 4 (also zoomed in Fig 3, Right) and this constitutes one of the key observations of the work we are reporting here. Under independence assumption, a given mode either uniformly (blindly) suppress or reinforce other modes or would not have a significant effect on other modes (other than mode 1 which has a direct interpretation as explained above). However, by taking a closer look at the power spectrum of the third mode we can easily observe that this mode is middle-powered and represents a wide range of frequencies, peaking slightly at the relatively high frequency (0.20 Hz including frequencies which have been usually associated with non-physiological sources of variations such as motion artefacts that are commonly filtered in BOLD analyses). On the other hand, mode 4 has a spectrum very similar to that of the BOLD signal, and the map at row 3 and column 4 illustrates an interesting suppression effect on this mode on a subset of networks in VIS, cognitive controls (CC) (including IPL) and DM (including R/L AG and ACC) by thigh mid-powered, higher-frequency mode 3. Commonly held beliefs about the nature of fMRI data would not suggest any dependency between these modes since these two are supposed to belong to very different sources of variation in captured signal [22–24].

Furthermore, through a regression analysis, we observed that cross-frequency dependence may vary between different groups of subjects. Specifically, we observed an age-correlated increase in the suppressive effect of networks in mode 1 on putamen and IPL when in mode 2 and, simultaneously, an age-correlated increase in the reinforcement effect of networks in mode 1 on putamen when in mode 4. This observation is interesting since the putamen is part of the sub-cortical domain of networks which is understood to be broadly connected to cortical networks [25] and these connections are believed to be involved in cognitive functions [26]. Although study of aging brain is out of scope of this paper (and the range of subject ages in this study is limited) such an observation highlights the potential benefits of incorporating cross-frequency dependence measures in connectivity-based aging brain studies. We also found that subject gender was associated with cross-frequency dependence, an interesting observation whose neuro-physiological interpretation is also beyond the scope of this paper.

Before concluding this section, we need to further clarify the actual nature of the instantaneous activities that each frequency mode is representing. The most important consideration in interpreting frequency modes is that each occurrence of a given mode is in fact representing instantaneous but “periodic” activities which consist of instantaneous activations and de-activations of the signal. The frequency spectrum of each mode represents the occurrence rate of these activations and de-activations. Consequently, a co-occurrence between two modes could be representing either positive or negative correlation since instantaneous activation of one signal could be aligned to either instantaneous activation (i.e. positive correlation) or de-activation (i.e. anti-correlation) of the other signal. In conclusion, reinforcement and suppression effects should not be associated with the commonly used correlation or anti-correlation measures. However, as the simulation results in S3 File suggest, there is an interesting correspondence between patterns of self-reinforcement of the individual modes and the patterns observed with conventional correlation analysis.

### Limitations and future works

As mentioned above, one of the major limitations of the proposed measure of cross-frequency dependence is its inability to estimate directionality of dependence in the way that correlation could measure in the form of positive and negative correlation. However, we believe that by investigating possible approaches to incorporate phase information in a possibly new measure of cross-mode dependence we would be able to measure the directionality as well.

The introduced method in this study, although enables to capture unique aspect of inter-network frequency-dependencies, cannot itself contribute toward interpreting the observed cross-frequency dependencies and their variations among different types of subjects. Our main goal here has been to introduce a novel way to measure dependence and connectivity among fMRI networks, and their transient spectral properties, which resulted in making new observations on the actual nature of the fMRI data. Also subject-wise variation of the measure establishes its advantage in designing new neurophysiological biomarkers in study of human brain. However, more complex designs such as multi-modal or task-based studies is needed to be able to make the observation more interpretable with respect to actual underlying neurophysiological brain activities. For example, there is a possible relation between cross-frequency connectivity observed in this study and the cross-frequency coupling observed in EEG or MEG studies. An interesting question would be if we could trace modulation of well-known frequency bands of neuronal oscillations in the frequency modes we showed here, A multi-modal study can be performed to shed light on this valid question as a future work.

Also, interpretation of our results is limited to the methods of our choice. For example, frequency modes, here, are defined as the clusters that are formed by k-means clustering which means the cluster assignments are hard assignments (i.e. only one frequency mode is assigned to a time-point) and also the shapes of the modes are limited to the inherited assumption of the k-means such as constant variance in each cluster. More complicated clustering such as non-parametric and fuzzy clustering can be investigate in future and reproducibility of the modes among different clustering can be studies.

Finally, we need to emphasize that we are not suggesting this method as a replacement for existing methods. In fact, we believe there are rich complementarities between cross-frequency and full-spectrum approaches to network connectivity that promise the kind of augmented effectiveness in clinical diagnostic settings already seen with combined use of static and dynamic connectivity measures[27].

## Supporting information

S1 File Spatial maps of ICA components as functional networks used in this study. (PDF)

S2 File Selecting number of frequency modes. (PDF)

S3 File Results of cross-frequency dependence with simulated time-courses based on conventional model of BOLD signal generation. (PDF)

S4 File Investigation of bias in the estimation of the *c*co-occurrence measure due to the limited number of available samples as well as possible bias due to the clustering labeling. (PDF)
