# Supplementary material for: Time-varying spectral power of resting-state fMRI networks reveal cross-frequency dependence in dynamic connectivity
Source: PLoS One. 2017 Feb 13;12(2):e0171647. doi: 10.1371/journal.pone.0171647 (PMC5305250; doi:10.1371/journal.pone.0171647)
Supplement: S1 File — (PDF) [file pone.0171647.s001.pdf]

S1 Spatial maps of ICA components as functional networks used in this study

In following we are showing spatial maps of all 50 ICA components identified as intrinsic connectivity networks. Sagittal, coronal and axial slices for each SM is shown. More detailed information on these ICNs can be find in supplementary material of [1].

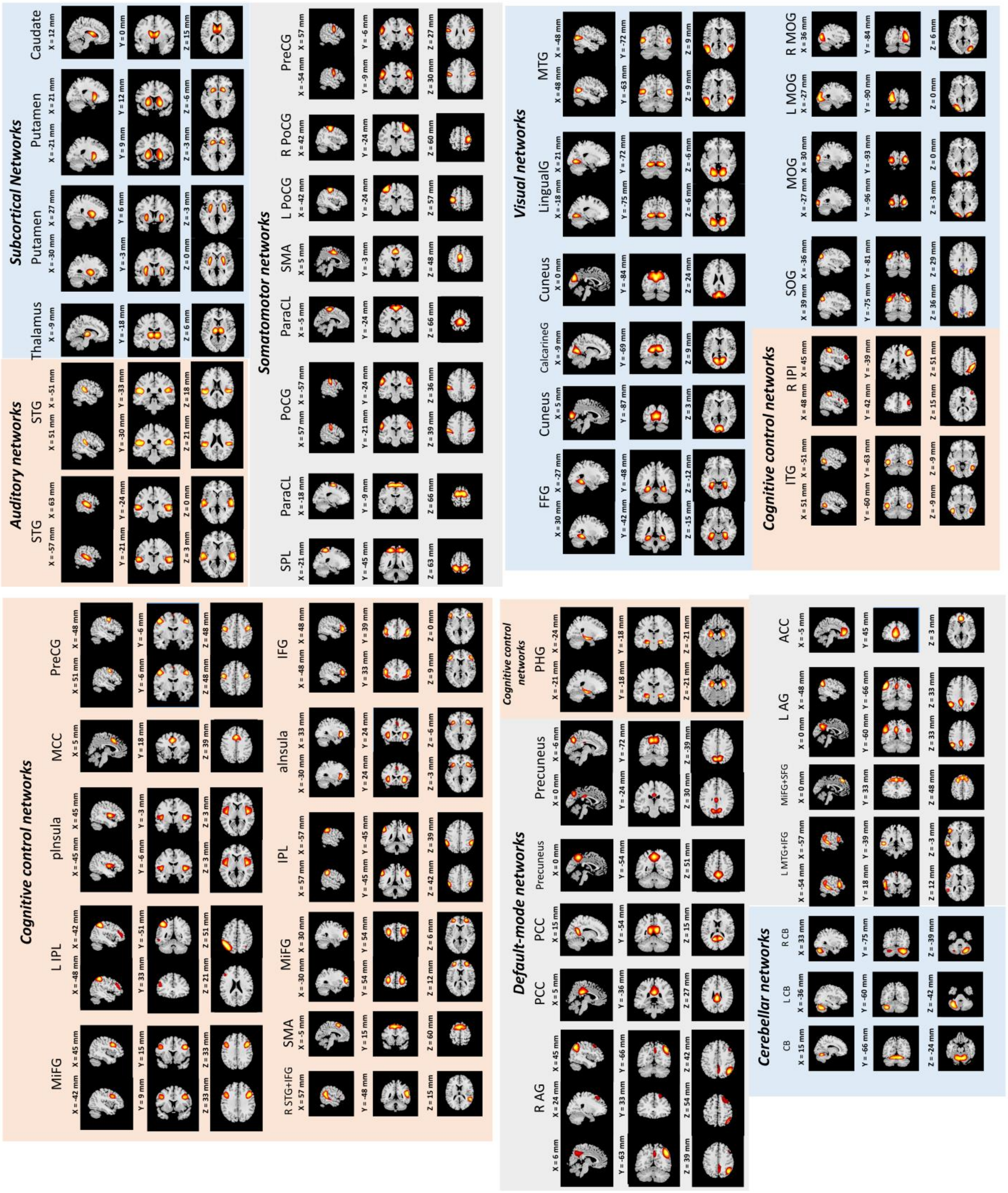

S 1 Figure: Sagittal, coronal and axial slices of SMs of ICA components that have been identified as ICNs and have been used in this study.

1. Allen EA, Damaraju E, Plis SM, Erhardt EB, Eichele T, Calhoun VD. Tracking Whole-Brain Connectivity Dynamics in the Resting State. Cerebral cortex. 2014;24(3):663-76. doi: DOI 10.1093/cercor/bhs352. PubMed PMID: WOS:000331845700010.
