# Supplementary material for: Time-varying spectral power of resting-state fMRI networks reveal cross-frequency dependence in dynamic connectivity
Source: PLoS One. 2017 Feb 13;12(2):e0171647. doi: 10.1371/journal.pone.0171647 (PMC5305250; doi:10.1371/journal.pone.0171647)
Supplement: S2 File — (PDF) [file pone.0171647.s002.pdf]

S2 Selecting number of frequency modes

K-mean clustering with different number of clusters was ran on the concatenated data of instantaneous frequency contents of all networks at all time-point for all the subjects and for each cluster results, ratio of variance in each cluster (inside cluster dispersion) to the variance between clusters (outside cluster dispersion) and the ‘k’ corresponding to the elbow of such curve is our selected number of clusters which here is ‘4’.

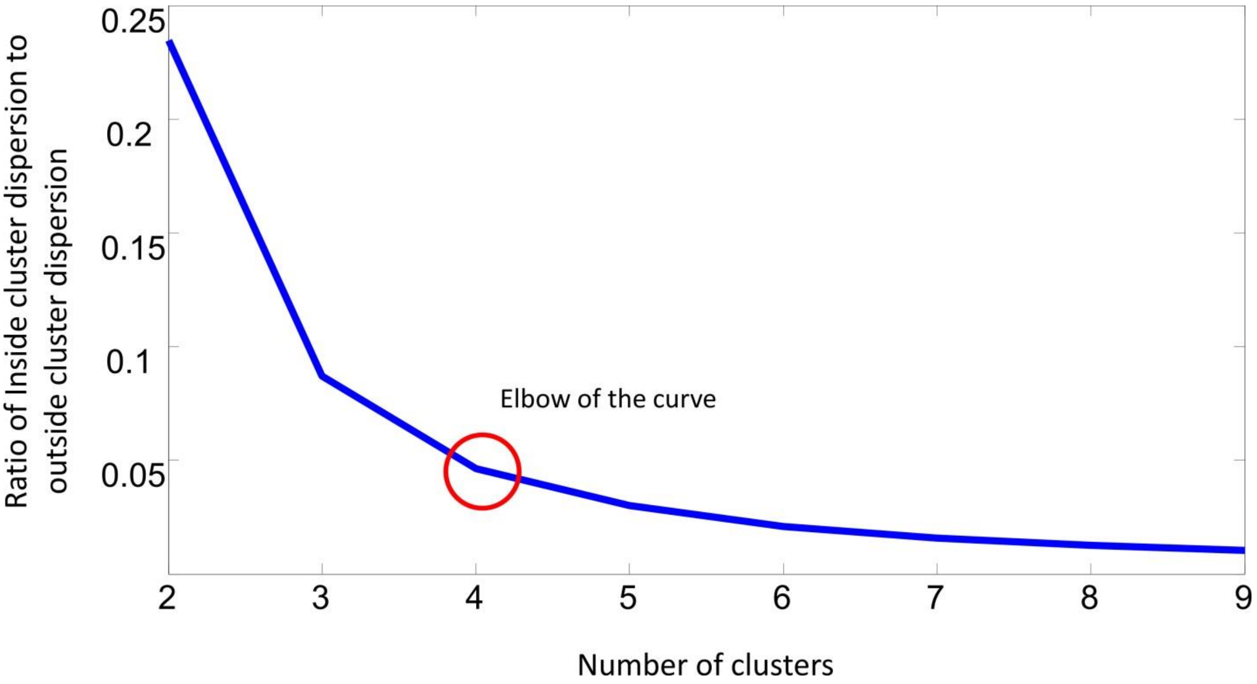

S 2 Figure: Elbow curve generated with running k-means clustering with different number of clusters.
