# Supplementary material for: Time-varying spectral power of resting-state fMRI networks reveal cross-frequency dependence in dynamic connectivity
Source: PLoS One. 2017 Feb 13;12(2):e0171647. doi: 10.1371/journal.pone.0171647 (PMC5305250; doi:10.1371/journal.pone.0171647)
Supplement: S3 File — (PDF) [file pone.0171647.s003.pdf]

S3 Results of cross-frequency dependence with simulated time-courses based on conventional model of BOLD signal generation

To emphasize significance of the results, we also investigate frequency modes of simulated time-courses based on conventional models of BOLD signal generation. We used SimTB toolbox [1] to simulate time-courses of 3 components of 100 subjects, modeled as the convolution of canonical model of hemodynamic response functions (HRF) (with randomized parameters for each subject and component) with series of randomly generated spikes. Probability of occurrence of a spike at a time-point is set to 0.3. Two of the time-courses were designed to have correlated spiked (0.8) by sampling from Dichotomized Gaussian distribution [2]. And finally a Gaussian random noise is added to the signal with standard deviation of 0.3 which makes the SNR of the simulated signal to be around 1.5. Note that this noise level is a conservative estimation of the actual SNR of the signal which we used in the study since the actual signal is in fact ICA-time courses which is a weighted average over voxel-wise time-series.

The rest of the procedure is the same as explained in the paper. First, four frequency modes shared between time-courses and subjected are captured S3 Fig A then, the cco-occurrence between the modes is estimated and is shown in S3 Fig B. As we can see from the figure, other than the self-reinforcement effect of mode 4 between correlated time-courses, there is no other evidences of dependence across frequency modes which, again, signify observed cross-frequency dependence in actual data.

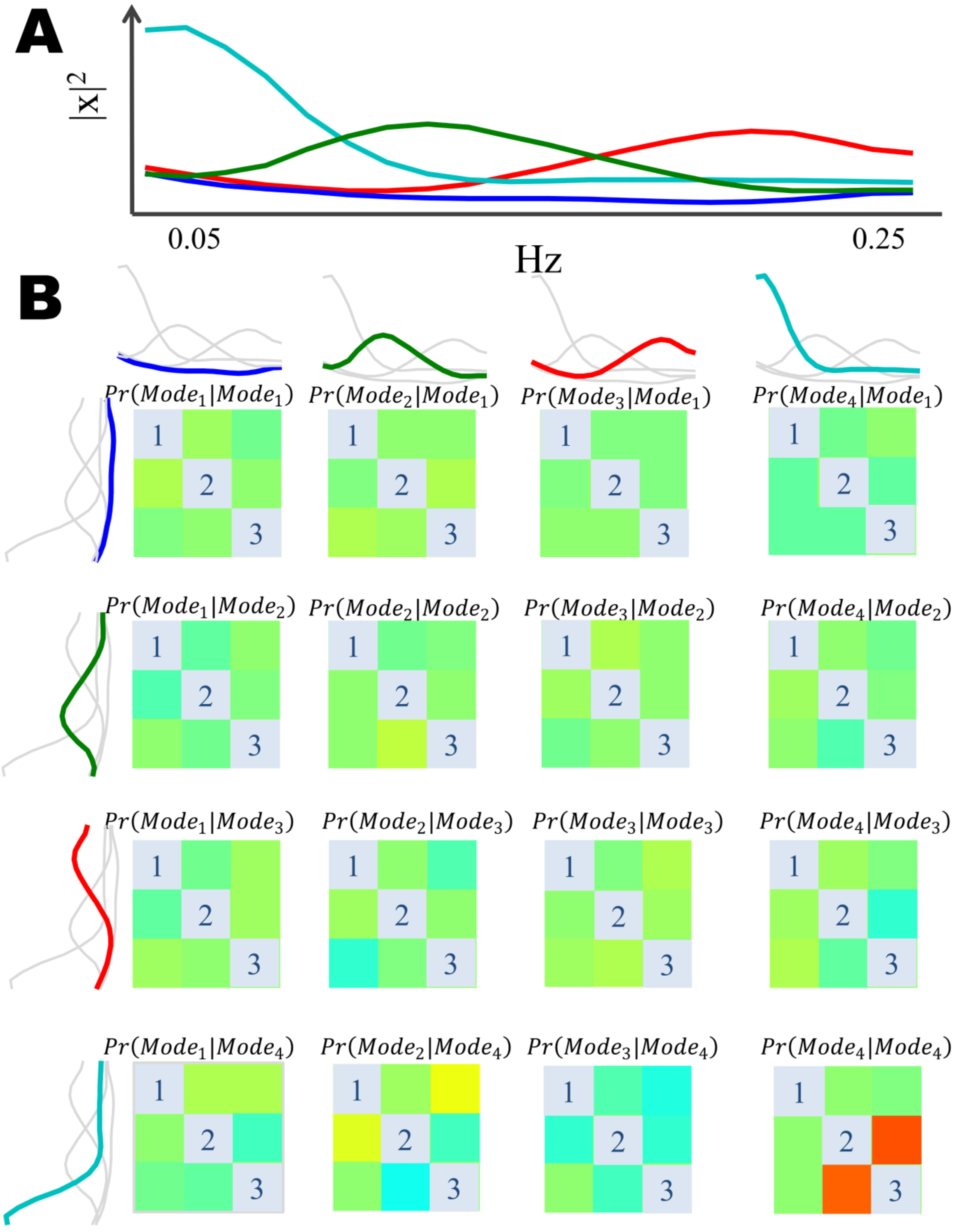

S 1 Figure: Simulation analysis.

(A) Frequency modes captured from simulated time-courses. (B) ccoccurence-maps of the frequency modes.

1. Erhardt EB, Allen EA, Wei YH, Eichele T, Calhoun VD. SimTB, a simulation toolbox for fMRI data under a model of spatiotemporal separability. Neuroimage. 2012;59(4):4160-7. doi: 10.1016/j.neuroimage.2011.11.088. PubMed PMID: WOS:000301090100106.
2. Macke JH, Berens P, Ecker AS, Tolias AS, Bethge M. Generating Spike Trains with Specified Correlation Coefficients. Neural computation. 2009;21(2):397-423. doi: DOI 10.1162/neco.2008.02-08-713. PubMed PMID: WOS:000263387000005.
