# Supplementary material for: Time-varying spectral power of resting-state fMRI networks reveal cross-frequency dependence in dynamic connectivity
Source: PLoS One. 2017 Feb 13;12(2):e0171647. doi: 10.1371/journal.pone.0171647 (PMC5305250; doi:10.1371/journal.pone.0171647)
Supplement: S4 File — (PDF) [file pone.0171647.s004.pdf]

S3 Investigation of bias in the estimation of the Cco-occurrence measure due to the limited number of available samples as well as possible bias due to the clustering labeling

Since co-occurrence is defined as the conditional probability of occurrence of event A given that event B has occurred ($\Pr(A|B = 1)$) the available samples to estimate this conditional probability is limited to the number of occurrences of even B (i.e. B=1) and if such assurance is rare, this might make estimation of the conditional probability less accurate. To investigate if in our study, occurrence rate of individual modes is biasing estimation of cco-occurrence of the modes, we conducted two separate simulations as follow:

For the first simulation, after the clustering step, we randomly and temporally shuffle cluster assignments of the time-points. Note that this does not change the individual occurrence rate of the mode but we remove any actual dependence between occurrence of the modes across networks. If we had an inaccurate estimation of the conditional co-occurrence between the modes due to the small individual occurrence of the modes, we would have expected to observe false dependence between the modes, however, our result suggests that this is not the case (Figure below).

With the first simulation, there is a chance of removing possible source of bias in the proposed measure of dependence due to the clustering itself such as smoothness of the assignment of the clusters. To address this, we conduct the second simulation in which instead of shuffling the clustering assignments of the time-point we estimate the cco-occurrence between the modes in different networks each belonging to different subjects. Since we are working with resting-state data we expect minimal dependence between time-courses of networks of different subjects and consequently minimal dependence between occurrences of the modes in different networks and between different subjects. Again the below figure rules out the biased estimation of cco-occurrence of the modes.

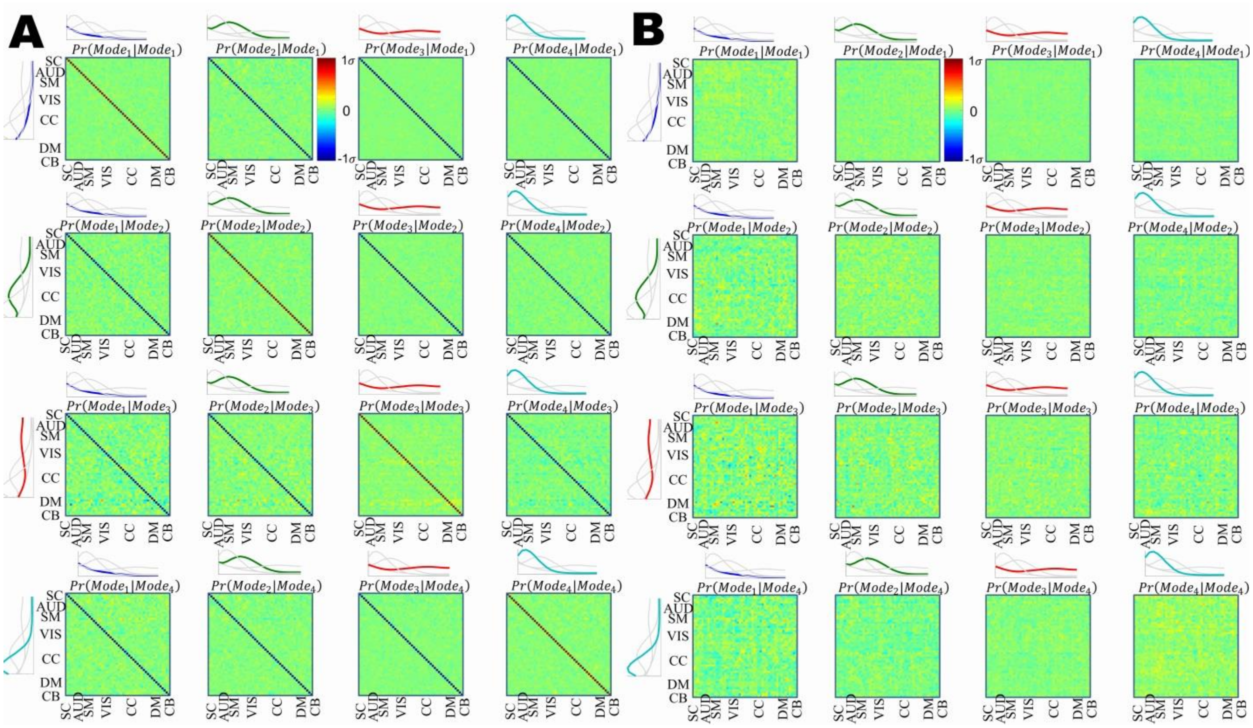

S 4 Figure: (A) Observed cco-occurrence between the modes under independence assumption forced by the shuffling in the first simulation. (B) Observed cco-occurrence between the modes while each network belongs to different subjects and consequently we expect minimal dependence.
